# Don’t Give-Up: Why some intervention schemes encourage suboptimal behavior

**DOI:** 10.3758/s13423-024-02537-w

**Published:** 2024-07-23

**Authors:** Doron Cohen, Yael Shavit, Kinneret Teodorescu

**Affiliations:** 1 Department of Psychology, University of Basel, Missionsstrasse 62a, 4055 Basel, Switzerland; 2 Faculty of Data and Decisions Sciences, Technion–Israel Institute of Technology, Haifa, Israel

**Keywords:** Behavioral economics; Behavioral change; Incentivization programs; Learning

## Abstract

Many social challenges stem from individuals’ tendency to prefer immediately rewarding but suboptimal behaviors (“Give-Up” options) over more costly endeavors that yield much better outcomes in the long run (“Try” options). For example, many people forgo the long-term benefits of formal education, healthy diets, learning new technologies, and even finding true love. This paper examines various incentivization programs that combine external rewards and punishments to discourage such counterproductive behaviors, which often result in only temporary behavioral change. Our findings suggest that some interventions’ limited impact may be due to their focus on only shifting behaviors from “Give-Up” (e.g., dropping out of college, avoiding the gym) to “Try” (e.g., attending college, exercising regularly), without promoting sufficient exploration of these “Try” options. Yet exploration of the long-term benefits of “Trying” may be crucial to increase the chances of long-term learning and commitment. Using a simplified abstraction of this dilemma, our results show a high tendency to choose “Give-Up” options prior to intervention. Examination of four different incentivization strategies suggests that only rewarding exploration of new “Try” options is a straightforward strategy to increase exploration and optimal choice. Punishing both the selection of “Give-Up” options and the choice to exploit suboptimal “Try” options produced similar results. Other common guidance strategies were less effective, as these strategies simply tended to replace one suboptimal behavior with another. Surprisingly, punishments seemed to be a relatively more successful incentive than rewards. We discuss how these insights can help guide policy aiming to improve long-term outcomes through incentivization.

**Supplementary information:**

The online version contains supplementary material available at 10.3758/s13423-024-02537-w.

## Introduction

Many social issues in education, work, finance, and health depend in part on individuals’ decisions to either try to maximize their long-term utility or settle for suboptimal options instead. For example, truancy (i.e., the intentional absence from compulsory education; Chang & Jordan, 2011) often reflects the choice to give up on efforts to maximize one’s potential in favor of more immediate but lesser rewards. Similarly, maintaining an unhealthy diet and opting for sedentary activities can reflect the decision to give up on pursuing a healthier lifestyle, settling for short-term enjoyments instead. Yet maintaining the costlier effort to try finding the best long-term strategies can reveal highly beneficial options, ensuring that trying, rather than giving up, is optimal in the long run.

In this paper, we focus on settings in which choice options can be categorized into two types: “Try” alternatives, which offer high rewards but are hard to obtain (e.g., pursuing a college degree, exercising, cooking at home), and “Give-Up” alternatives, which provide lower but more immediate rewards (e.g., watching television instead of attending class). Previous research suggests that in binary choice settings, individuals often prefer “Give-Up” options that provide immediate gratification over “Try” options that are much better in the long run. That is, people are predicted to avoid the superior “Try” options when these offer higher outcomes with either a low probability (also known as underweighting of rare events, e.g., Camilleri & Newell, 2011; Ert & Erev, 2017; Hertwig et al., 2004; Lejarraga & Hertwig, 2021; Teodorescu et al., 2013) or a temporal delay (also known as temporal discounting; e.g., Dai & Busemeyer, 2014; Shavit et al., 2022). In line with these findings, numerous incentivization programs have been introduced to augment the outcomes from “Try” alternatives, to reduce the likelihood people give up on maximizing their long-term rewards.

Specifically, to help people modify their behavior for better long-term outcomes, interventions and policy programs often employ external incentives. Examples include programs aimed at promoting healthy eating (Shavit et al., 2021; Wall et al., 2006), increasing gym attendance (Charness & Gneezy, 2009; Milkman et al., 2021; Mitchell et al., 2013), and boosting college enrollment and completion rates (Sjoquist & Winters, 2015a). However, the effectiveness of these programs is often short-lived, as behaviors tend to revert once the incentives are withdrawn (e.g., Acland & Levy, 2015; Beshears et al., 2021; Carrera et al., 2018; DeFulio & Silverman, 2012; Gneezy et al., 2011; John et al., 2011; Milkman et al., 2014; Royer et al., 2015).

We suggest that the failure of many interventions to effect lasting change may be due to their exclusive focus on using incentives to shift individuals from “Give-Up” to “Try” behaviors. By concentrating solely on this transition, such interventions often overlook the critical need to encourage exploration within the “Try” category, which is essential for long-term learning and retention. Specifically, finding highly rewarding “Try” options typically requires significant exploration costs, involving investment of time, effort, and other resources (see Mehlhorn et al., 2015). Moreover, since highly rewarding “Try” options are often rare, people are also likely to underweight the chance of finding them, leading to a tendency to underexplore the set of “Try” alternatives (e.g., Cohen & Teodorescu, 2021; Teodorescu & Erev, 2014a, 2014b).

For example, even if an intervention succeeds in incentivizing absent students to attend school, students might still underexplore effective learning strategies and engage in suboptimal behaviors (e.g., using their phones or conversing with peers during class). Similarly, rewarding children for each book read might encourage them to choose familiar, less challenging books with larger print (Schwartz, 1982). In such cases, favorable intervention effects might dissipate once the incentives are removed (Eisenberger & Cameron, 1996), as readers fail to discover great books that they will be intrinsically motivated to read. To counter such counterproductive behaviors, and to increase long-term benefits, interventions should promote both adoption of desired “Try” behaviors and exploration among “Try” alternatives. Enhancing exploration increases the likelihood that individuals discover engaging alternatives and may decrease the chance they will revert to less productive behaviors after incentives end. The following study clarifies this observation.

**Fig. 1:**
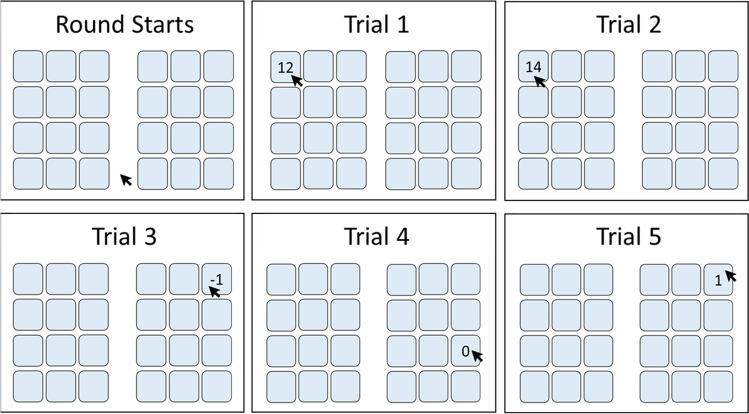
Example screenshots of the “Try or Give-Up” task. The figure presents screenshots of five hypothetical trials. In each trial, the participant must choose one key among the 12 × 2 available keys, sorted into either a “Try” or a “Give-Up” matrix (participants were not informed about the specific nature of each matrix). See Table 1 for the complete incentive structure

We focus on the experimental choice setting presented in Fig. 1 to compare and quantify the effectiveness of different interventions in overcoming suboptimal behavior in a controlled, context-free setting. In this setting, participants repeatedly choose between two categories of alternatives: One representing “Give-Up” alternatives that yield small but certain rewards, and another representing “Try” alternatives that are usually costlier but sometimes exceptionally rewarding. We hypothesized (https://aspredicted.org/H5V_77Q) that interventions focusing solely on shifting participants from “Give-Up” to “Try” categories, without addressing under-exploration within the “Try” alternatives, would fail to create a lasting positive effect.

Our analysis examines how individuals choose between and within the two classes of options—”Try” and “Give-Up”—in an abstract environment. This approach allows us to focus on the dynamics of learning while excluding other factors such as structural and social barriers (e.g., Dittmar, 2015; Johns, 2013; Melamed, 1995). Although far removed from real-world complexities, using abstract choice tasks to explore and test incentivization strategies offers clear advantages, providing insights that are difficult to obtain in field studies. This method helps isolate the effects of the incentive structure by controlling for motivational factors. This control enables a precise analysis of how incentives and learning influence two types of suboptimal behaviors over time: Choosing “Give-Up” options, and under-exploration among “Try” options. Further, our method allows comparing the efficacy of punishments and rewards in addressing suboptimal behaviors. Given the challenges of implementing punishment-based programs in real-life settings (e.g., Evers et al., 2017; Harring, 2016; Tannenbaum et al., 2013), insights from such controlled experiments are particularly valuable.

## Methods

### Participants

A power analysis (available in the project’s OSF depository) based on a pilot study indicated that a sample size of 53 participants in each condition is suitable. We recruited 216 participants (49% identify as females, 50% as males, *M*_age_ = 38.7 years, *SD*_age_ = 11.5, range: 18–60) from Prolific Academic (https://prolific.ac), randomly allocated to four between-subject conditions. Participants were informed they will earn a fixed show-up fee of £2.4 (about $3), and a bonus based on their choices (see Procedure for payoff rule). The mean bonus was about £0.16 (about $0.2). The experimental session lasted 23 min. on average.

### Materials and procedure

Participants played three experimental blocks successively. Each block comprised 15 rounds, and each round included 12 trials. In each round, two matrices of keys appeared on-screen, each matrix displaying 12 (3 × 4) unmarked keys. In each trial, the participant’s task was to choose one key from either of the matrices. After a choice was made, the outcome from that choice appeared on the selected key for 1 s (see Fig. 1). Participants were incentivized to maximize their earnings in each round: The average of the accumulated earnings in three randomly selected rounds, one from each experimental block, determined participant’s bonus payment. Participants were informed they will play three experimental blocks one after another, for 15 rounds each with 12 trials in each round. No other information was given regarding the exact payoff structure in each of the experimental rounds.

Table 1 presents the task parameters (i.e., the incentive structure) used for each experimental block and condition. After a specific key was first pressed in each round, its outcome remained set until the end of that round. The location and outcome of each key elapsed when a new round began. Each matrix was defined by a different distribution of potential outcomes. One matrix represented the class of “Give-Up” behaviors (hence, the Give-Up matrix), in which each key either yielded a positive outcome of + 1 or + 2 points with equal probability. The second matrix represented the class of “Try” behaviors (hence, the Try matrix) that yielded an outcome of + 1 with probability 0.9, otherwise yielding an outcome of + 14. The location of the matrices on participant’s screen was counterbalanced and remained constant throughout the experiment.

**Table 1:** Experimental incentive design by experimental blocks and treatment conditions

		Give-Up		Try		Exploration costs
Treatment	Reward Type	Outcome	*p*	Outcome	*p*	
Baseline Game (Blocks #1 and #3)	*Low*	+ 1	.5	+ 1	.9	− 2
	*High*	+ 2	.5	+ 14	.1	− 2
*Block #2 (random allocation)*						
− GiveUp: Punish any Give-Up	*Low*	− 1	.5	+ 1	.9	− 2
	*High*	0	.5	+ 14	.1	− 2
− GiveUp& − ${{\varvec{T}}{\varvec{r}}{\varvec{y}}}_{{\varvec{L}}{\varvec{o}}{\varvec{w}}}$: Punish any Give-Up & Low Try choice	*Low*	− 1	.5	− 1	.9	− 2
	*High*	0	.5	+ 14	.1	− 2
+ Try: Reward any Try	*Low*	+ 1	.5	+ 3	.9	− 2
	*High*	+ 2	.5	+ 16	.1	− 2
+ Try_Explore_: Reward Try exploration	*Low*	+ 1	.5	+ 1	.9	0
	*High*	+ 2	.5	+ 14	.1	0

Each row represents the parameters of one of the three experimental blocks. Each participant faced the Baseline treatment (first row) for the first and third blocks. Participants were randomly allocated to one of the other four treatment conditions played as the second block. The Try and Give-Up matrices were composed of 12 keys of either *Low* or *High* outcome keys appearing with probability *p*. ${Try}_{Low}$ refers to exploitation of *Low* options in the Try matrix. Exploration costs represent the points subtracted when choosing a specific alternative (key) for the first time in each round

Building on previous research that highlights the impact of exploration costs in real-life exploration–exploitation dilemmas (e.g., Mehlhorn et al., 2015; Teodorescu & Erev, 2014a, 2014b), we incorporated a specific cost for exploring new options in our experiment. Specifically, an exploration cost of 2 points was deducted from the participant’s score each time a key was pressed for the first time in a round. No exploration costs were applied to subsequent selections of the same key (i.e., when exploiting a familiar option). Once selected, the outcome associated with a key remained constant for the duration of that round.

The current experimental setup defines two types of suboptimal behaviors. The first is selection of Give-Up options, which usually give a better outcome (+ 2) than most Try options (+ 1), but are suboptimal in the long run (as Try options can also yield a better outcome of + 14). A second type of suboptimal behavior emerges in the Try matrix when suboptimally exploiting the frequent + 1 options rather than trying to find the best + 14 options. That is, choosing to exploit a familiar, low-outcome key and avoiding exploration costs, rather than exploring for the rare (+ 14) options. Note that these suboptimal behaviors reflect two local maxima (i.e., options that offer a higher value than most other immediate alternatives but a lower value than the best available alternatives; Yechiam et al., 2001). Give-Up options define the first local maximum, while exploitation of + 1 Try alternatives (referred to as ${Try}_{Low}$ alternatives) defines the second.

To gain more insight into participants’ learning and performance, we compare their choices with a benchmark for optimal behavior in the current task. Appendix A includes a simulated analysis comparing various strategies to this optimal benchmark (R code available at https://osf.io/w3vea). The optimal strategy in this game is dynamic: First, one must start each round by searching for a + 14 key (in the Try matrix), and once found, exploit that key to the end of that round. Yet if a high-outcome key is not found after searching for at least nine trials, the optimal strategy changes (see Wilson et al., 2014, for the dependence of optimal choice on number of remaining choices). Specifically, instead of continuing to search for a + 14 option, the optimal strategy is to shift to the Give-Up matrix and search instead for a + 2 key. Once a + 2 key is found, one should exploit it to the round’s end. Importantly, participants were not provided with information about the incentive structure and could only learn about it through feedback and experience with the task. Thus, the optimal solution, which assumes full information, can serve only as a benchmark for optimal behavior after learning has occurred.

To ensure the theoretical validity of our conclusions, we report here analyses including only choices made within the first nine trials (https://aspredicted.org/H5V_77Q). Figure B2 (Appendix B) presents the observed choice rates over all 12 trials. An analysis like the one presented below but including all 12 trials suggests that the results are essentially the same (see Online Supplementary at https://osf.io/w3vea).

Participants were randomly allocated to one of four treatment conditions. In each condition, participants played three experimental blocks (with 15 rounds in each) in the following order. First, a pretreatment baseline experimental block was played by participants in all conditions, which included the baseline payoff structure explained above. In each condition, a different incentivization intervention was implemented in the second experimental treatment block (and only in that block), with an additional punishment or reward scheme meant to increase optimal choice rates. Following the treatment block, a posttreatment block, with the treatment incentives removed, was played last. That is, in the first (pretreatment) and last (posttreatment) experimental blocks, all participants were presented with the same payoff structure.

We implemented four treatment conditions in the current study that simulate different intervention programs to increase optimal choices. As noted, in all treatment conditions, the external incentives were implemented only in the second experimental block. In condition “punish Give-Up choice” (hence − GiveUp), a 2-point penalty was imposed on any outcome derived from choosing Give-Up alternatives (e.g., punishing truancy by withdrawing student’s home privileges). In condition “punish both Give-Up and suboptimal Try” (hence − GiveUp& − ${Try}_{Low}$), a 2-point penalty was imposed on any choice that does not correspond to the optimal strategy (i.e., searching for ${Try}_{High}$ keys; see Table 1). In condition “reward any Try choices” (hence + Try), a 2-point reward was added to any choice of an alternative in the Try set (e.g., rewarding students for showing up to class). In condition “reward Try exploration” (hence + Try_Explore_), a 2-point reward was added to any choice of an alternative in the Try set when it was chosen for the first time (negating exploration costs for Try alternatives). We had 53, 55, 54, and 54 participants in conditions − GiveUp, − GiveUp& − ${Try}_{Low}$, + Try and + Try_Explore_, respectively.

We preregistered the following hypotheses: We hypothesized that the most effective guidance strategies involving rewards and/or punishments will be those leading people to explore new “Try” options. In contrast, interventions which generally promote selection of “Try” options (without a specific emphasis on exploration) over “Give-Up” options will be less effective due to replacement of one suboptimal behavior with another. Specifically, we predicted that Treatments − GiveUp&−${Try}_{Low}$ and + Try_Explore_ will lead to higher Optimal rates and lower choice rates of Give-Up options and ${Try}_{Low}$ exploitation, compared to − GiveUp and + Try conditions.

#### Dependent variables (DV)

We limit the current analysis to only choices made within the first nine trials, as the optimal strategy benchmark changes after that point (see Appendix A). We focus our analysis on the following dependent variables: (1) choice rates of options in the Give-Up matrix, (2) under-exploration of Try options, or ${Try}_{Low}$ exploitation rates (choosing familiar + 1 outcomes in the Try matrix), and (3) choices in line with the optimal benchmark (i.e., looking for a ${Try}_{High}$ outcome [+ 14] and exploiting it once found).

#### Preregistered analysis plan

We planned two one-way multivariate analyses of variance (MANOVAs) on the three dependent variables (Give-Up, ${Try}_{Low}$ exploitation rates, and optimal choice rates), with treatment (1–4, between-subjects) as the independent variable. Because Give-Up rates were strongly correlated with optimal choice rates in all four treatment conditions (${\overline{r} }_{Fisher Z}= -0.961$), we decided to exclude these from the MANOVA analysis (as this violates the assumption of absence of multicollinearity/singularity; Tabachnick et al., 2013). Thus, our MANOVAs include only two dependent variables: ${Try}_{Low}$ exploitation rates and Optimal choice rates. Including Give-Up rates in the MANOVAs does not change the results in any significant way. Table 2 presents descriptive statistics for the calculated difference scores used in the analysis presented below.

**Table 2:** Difference in choice rates and payoffs as treatment and retention effects

	Treatment conditions			
Dependent Variable	− GiveUp: Punish any Give-Up	− GiveUp& − ${{\varvec{T}}{\varvec{r}}{\varvec{y}}}_{{\varvec{L}}{\varvec{o}}{\varvec{w}}}$: Punish any Give-Up & Low Try choice	+ Try: Reward any Try	+ Try_Explore_: Reward Try exploration
*% Change: 2nd treatment block* − *1st pretreatment baseline block [95% CI]*				
Give-Up	− 24 [− 30, − 17]	− 24 [− 29, − 18]	− 22 [− 27, − 16]	− 22 [− 28, − 17]
${Try}_{Low}$(exploiting + 1 in Try)	4 [1, 7]	0.2 [− 1, 1]	12 [7, 16]	0.4 [− 2, 2]
Optimal	20 [13, 27]	24 [18, 29]	11 [5, 17]	23 [16, 29]
Mean payoffs (excluding treatment effects)	1.2 [0.6, 1.7]	0.9 [0.4, 1.5]	0.9 [0.3, 1.4]	1.5 [1.0, 2.0]
*% Change: 3rd posttreatment Block* − *1st baseline block [95% CI]*				
Give-Up	− 14 [− 22, − 7]	− 18 [− 25, − 12]	− 2 [− 11, 6]	− 11 [− 19, − 3]
${Try}_{Low}$(exploiting + 1 in Try)	0.4 [− 1, 2]	− 1 [− 1, 1]	2 [1, 4]	− 1 [− 2, 2]
Optimal	15 [7, 22]	19 [13, 26]	1 [− 7, 10]	11 [3, 20]
Mean payoffs	1.2 [0.6, 1.7]	1.1 [0.6, 1.7]	0.8 [0.2, 1.4]	0.9 [0.3, 1.5]

Each column shows the difference scores for each dependent variable across participants. Negative values denote a decrease, and positive values imply an increase between the second treatment block and first baseline block (middle rows) and between the third posttreatment block and first baseline block (bottom rows). Each cell shows the % difference and 95% CI across participants in each treatment condition. See Table 1 for experimental design and definition of treatment conditions and Methods for description of dependent variables. Mean payoffs are the average outcome per trial

The first MANOVA predicts short-term improvement: The difference (for each DV) between the second and first blocks. The second MANOVA predicts retention: The difference (for each DV) between the third and first blocks. Follow-up univariate ANOVAs were performed for all sources of variation if statistically significant multivariate differences were found. We also ran two univariate ANOVAs, with treatment (1–4, between-subjects) as the independent variable, and the differences in payoffs earned between the second and first blocks and third and first blocks as dependent variables. Finally, we report here a similar analysis of payoffs earned between the second and first blocks that includes as dependent variable the “baseline” payoffs as DV, calculated by removing treatment-specific punishments and rewards (i.e., assuming the baseline payoff structure). The latter analysis ensures comparability between treatment effects.

## Results

**Fig. 2:**
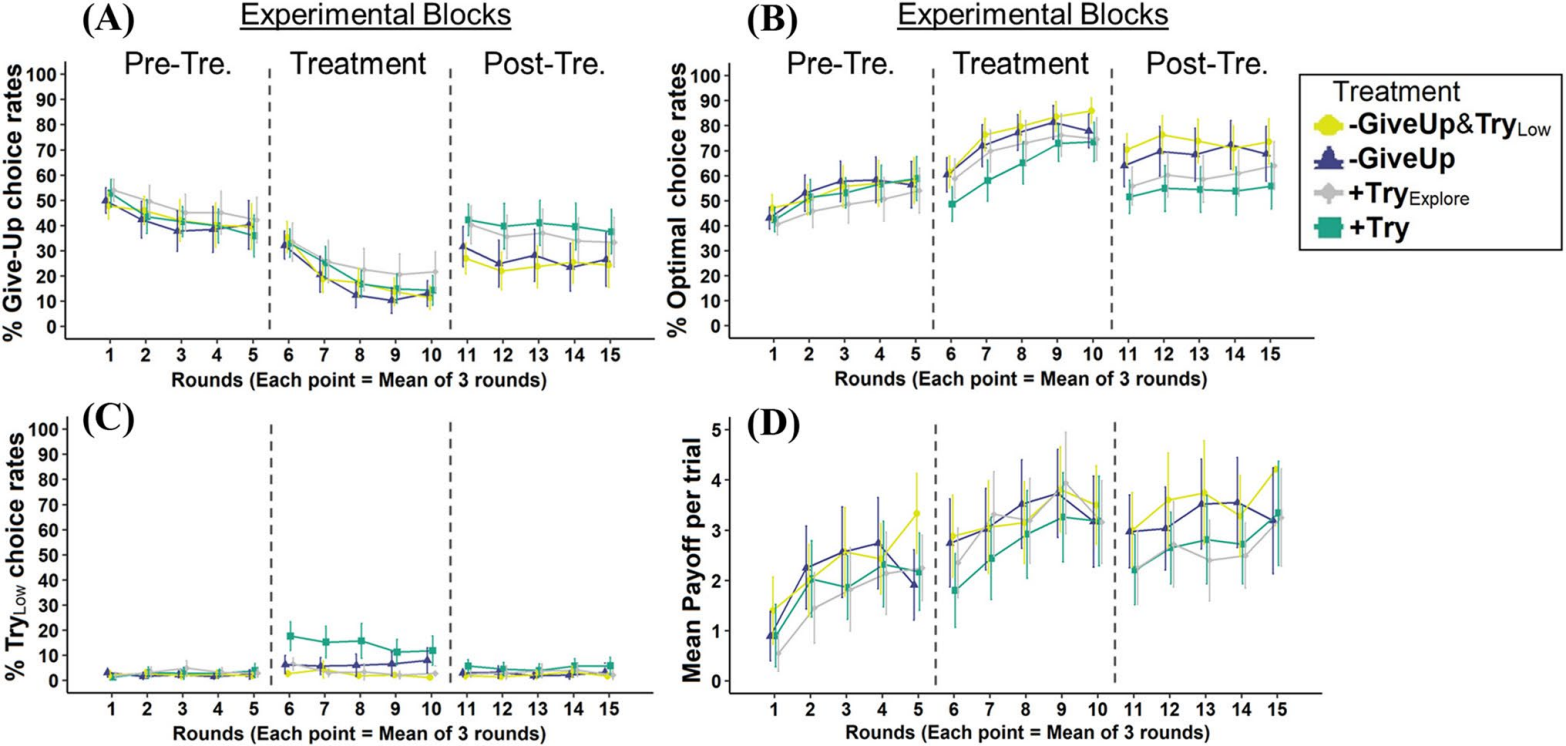
Summary of main results as a function of treatment and round*.* Each panel shows the main results averaged over the experimental rounds. Each point shows the mean of 3 rounds. Pre-Tre. and Post-Tre. titles denote the pretreatment (baseline) and posttreatment experimental blocks, respectively. **A** Give-Up rates. **B** Optimal (benchmark) strategy rates (finding and exploiting + 14 outcomes). **C** ${Try}_{Low}$ rates (choosing familiar + 1 options in the Try matrix). **D** Mean payoff per trial (in points). All figures exclude Trials 10–12. Mean payoff per trial calculation (**D**) excludes treatment-specific incentives. Error bars show 95% confidence intervals. See Appendix B for individual differences and within-round results. (Color figure online)

Figure 2 summarizes the main results over the experimental rounds and treatment conditions. Table 2 presents the difference scores on each dependent variable between the baseline block and subsequent rounds (following our preregistered analysis plan). Both summaries exclude Trials 10–12. We calculate the difference scores focusing either on treatment effects (treatment block – first baseline block) and retention (posttreatment − pretreatment baseline blocks). A positive (negative) difference score represents a relative increase (decrease) in the dependent variable compared with the (pretreatment) baseline control. An effective treatment should increase optimal choice rates while decreasing the Give-Up and ${Try}_{Low}$ exploitation rates. Figures B1 and B2 in Appendix B shows individual-level averages over the dependent variables and average choice rates as a function of trial number, respectively, across the different treatments and experimental blocks.

The first MANOVA (including differences on the target measures between the second- and first-round blocks) includes ${Try}_{Low}$ exploitation rates and optimal choice rates as dependent variable and treatment (four levels) as fixed effect (see Analysis plan for details). This analysis shows statistically significant difference between the four treatment conditions, *F*(6, 422) = 6.04, *p* < 0.001. Follow-up univariate ANOVAs shows this difference was significant for both ${Try}_{Low}$ exploitation rates, *F*(3, 211) = 12.79, *p* < 0.001, and optimal choice rates, *F*(3, 211) = 3.47, *p* = 0.017. There was no significant difference for Give-Up rates, *F*(3, 211) = 0.13, *p* = 0.944 (i.e., participants reduced Give-Up rates on the second block similarly in all four conditions). Follow-up post hoc analyses show the effect is driven by the + Try Treatment: ${Try}_{Low}$ exploitation rates were significantly higher in the + Try Treatment compared with the − GiveUp, *t*(211) = 3.44, *p* < 0.001; − GiveUp&−${Try}_{Low},$ *t*(211) = 5.40, *p* < 0.001; and + Try_Explore_, *t*(211) = 5.32, *p* < 0.001, conditions. In addition, optimal choice rates were significantly lower in the + Try Treatment compared with the − GiveUp, *t*(211) =  − 2.04, *p* = 0.042, − GiveUp& − ${Try}_{Low}$, *t*(211) =  − 2.91, *p* = 0.004, and + Try_Explore_, *t*(211) =  − 2.64, *p* = 0.009 conditions. No significant pairwise differences were observed between the other conditions (see Table 2). Thus, our results suggest the + Try treatment led participants to under-explore the Try matrix and encouraged choice of suboptimal Try (${Try}_{Low}$) options.

The second MANOVA includes the differences in the target measures between the third-and first-round blocks (see Fig. 2 and Table 2). This analysis shows statistically significant differences between the four treatment conditions, *F*(6, 422) = 2.31, *p* = 0.033. Follow-up univariate ANOVAs show this difference was significant for optimal choice rates, *F*(3, 211) = 3.77, *p* = 0.011, but not for ${Try}_{Low}$ exploitation rates, *F*(3, 211) = 1.77, *p* = 0.155. The difference for Give-Up rates was statistically significant, *F*(3, 211) = 3.24, *p* = 0.023. Follow-up post hoc analyses (focusing on optimal choice rates) show the statistical significance is driven by the + Try treatment: Optimal choice rates were significantly lower in the + Try treatment compared with the − GiveUp, *t*(211) =  − 2.40, *p* = 0.017, − GiveUp& − ${Try}_{Low}$, *t*(211) =  − 3.23, *p* = 0.001, but not to the + Try_Explore_, *t*(211) =  − 1.83, *p* = 0.068, conditions. No other significant pairwise differences were observed between the conditions. This suggests that incentivizing to choose any Try option (i.e., the + Try treatment) impaired participant’s performance on the third (posttreatment) block, as participants chose significantly more Give-Up options and explored the Try matrix significantly less.

Next, we analyze the average payoffs participants gained in the experiment. Figure 2 (Panel D) and Table 2 show the average payoff differences per round when controlling for the added treatment-specific rewards and punishments in each treatment. This analysis shows a positive impact on participant’s payoffs in all four treatment conditions: While the first block’s average payoff per trial across the four conditions was 1.98 points (95% CI [1.75, 2.20]), the second block’s average per trial payoff was 3.11 points (95% CI [2.86, 3.35], ~ 60% improvement). On the third block, the average per round payoff was 2.99 points (95% CI [2.73, 3.25], ~ 50% improvement over the first block). We find no statistical effect of treatment on the average per round (baseline) payoffs between the second and first blocks, *F*(3, 211) = 1.34, *p* = 0.261, or between the third and first blocks, *F*(3, 211) = 0.35, *p* = 0.791.

## Discussion

We evaluated the impact of different incentivization schemes using a simplified experimental exploration–exploitation task. The interventions were designed to encourage individuals to try finding challenging but beneficial alternatives, rather than giving up and settling for less rewarding options instead. The participant’s task was to repeatedly choose between “Give-Up” alternatives that yielded more certain but small rewards and “Try” alternatives that are usually costlier but occasionally very rewarding (and optimal in the long run). Our results suggest that while carefully designed incentivization schemes can have a positive impact on individual’s effort to find challenging but optimal options, some schemes may produce unwanted results. Specifically, we demonstrate how a frequently employed incentivization scheme that rewards choice of more challenging options, but does not eliminate exploration costs or reduce opportunity costs from Try options, can encourage undesirable behaviors (e.g., excessive exploitation of suboptimal options). Such undesirable behaviors manifested in the “ + Try” treatment, which rewards any selection of Try alternatives, as participants tended to under-explore the Try options, preferring to exploit suboptimal Try alternatives instead. This suboptimal behavior persisted even after incentivization was removed.

Although participant’s average payoffs did not show welfare loss in our brief experiment (see Figs. 2, B1, and B2), the examples surveyed below indicate that welfare loss from preference for suboptimal behavior can be significant. Specifically, incentive schemes that do not also encourage exploration of Try options might lead decision makers to settle for the least costly option that fulfills the requirements of the external incentive scheme. This, in turn, might increase the chance that decision makers never learn the value of exploration. Once external incentives are removed, suboptimal exploitation of Try alternatives is likely to be replaced by more immediately rewarding alternatives, such as those that imply giving up trying altogether. To address this challenge, we suggest that interventions need to focus on motivating individuals to explore a wide range of the endorsed options. By encouraging exploration within the domain of the desired behavior, the chances of discovering valuable but rare options increase. That, in turn, would provide individuals with more appealing alternatives to replace the counterproductive options they may have otherwise preferred. This insight highlights the need for careful consideration of the nuanced impact of different incentivization programs in specific contexts. We suggest such consideration can be aided by use of controlled laboratory tasks, which isolate the effect of the baseline incentive structure, as presented in the current paper.

Our results are in line with the success of exploration-encouraging interventions in the domain of nutrition and skill acquisition. For example, Shavit et al. (2021) show that incentivization schemes specifically designed to increase exploration of new options can significantly encourage healthier eating habits. This positive effect persisted for a year postintervention, in contrast to a control group in which any healthy choice was rewarded (like our + Try condition), that showed no such improvement. Similarly, complex skill acquisition protocols known as “emphasis change” (that focus on shifting trainee priorities during task practice; Gopher, 1993; Gopher et al., 1989, 2007) enhanced exploration and subsequently improved skill acquisition performance compared with more traditional training methods that did not encourage exploration (Cohen & Teodorescu, 2021; Gopher et al., 1989).

Another noteworthy example is the merit-based HOPE (Helping Outstanding Pupils Educationally; Zhang, 2011) program. The HOPE program awards students who meet and maintain some merit requirement (e.g., maintain a certain GPA average) with a college scholarship. HOPE, and similar programs, have been shown to significantly increase college enrollment rates (e.g., Cornwell et al., 2006; Sjoquist & Winters, 2015a), successfully encouraging students to try costly but ultimately beneficial alternatives. Yet studies also show that such interventions can backfire: For example, merit-based scholarships have been shown to decrease the likelihood a scholarship awardee will choose to major in STEM fields (Cornwell et al., 2006; Sjoquist & Winters, 2015b; Zhang, 2011). One explanation is that merit-based incentive programs typically require a certain GPA average for renewal, which in STEM areas might be harder to maintain. Such criterion for receiving financial aid makes exploration of the educational environment costly, a fact that might reduce student’s effort to explore that environment for valuable lessons and skills (Teodorescu & Erev, 2014b). According to our analysis, these merit-based incentives were successful in reducing the appeal of one type of suboptimal behavior (i.e., giving up on attaining a college degree). Yet it is possible that such programs neglected another type of suboptimal behavior (i.e., avoiding exploration costs and settling for less optimal options), as the decrease in STEM majors implies.

Our study also yielded a surprising result: Punishing behaviors associated with selection of “Give-Up” alternatives tended to be more effective than rewarding the selection of “Try” options. This is surprising, as previous research typically highlights punishment’s detrimental effects on exploratory behaviors (e.g., Ashby & Teodorescu, 2019; Cohen & Erev, 2021; Galea et al., 2015; Heininga et al., 2017; Kubanek et al., 2015; LaVigna & Donnellan, 1986; Maag, 2001; Skinner, 1965; Teodorescu & Erev, 2014a, 2014b; Wächter et al., 2009; Yin et al., 2023). Yet we note a key difference with the current study: Whereas in prior studies the applied punishment harmed rewards from exploration, our interventions punished suboptimal exploitation decisions. In the current study, punishing suboptimal exploitation directly targeted people who engaged in suboptimal behavior to begin with. In contrast, to be rewarded for optimal behavior required individuals to already engage in some optimal choices to get rewards. This interaction between individual differences in baseline behavior and the effects of punishment vs. reward is illustrated in Fig. B1.

An additional potential contributor to the surprising success of punishments in the current task is the idea of “loss-attention”: The argument that experiences of losses increase one’s sensitivity to task incentives (Yechiam & Hochman, 2013). Notably, although punishing selection of Give-Up options (i.e., the − GiveUp treatment) was effective in improving people’s performance, it also significantly increased suboptimal exploitation of Try options (${Try}_{Low}$; see Table 2). These results highlight the need for additional research to shed light on the interaction between exploration and punishment in Try or Give-Up dilemmas.

Our study also suggests a possible alternative explanation for the behavioral phenomenon known as motivational crowding out (Frey & Jegen, 2001). Motivational crowding out implies that attaching positive and/or negative monetary incentives to a behavior reduces intrinsic motivation to perform that behavior (Festré & Garrouste, 2015; Frey & Jegen, 2001). This is said to happen when initial engagement and performance levels deteriorate once incentives are removed, compared with the same levels prior to the incentivization period (Gneezy et al., 2011). Yet it is not easy to measure intrinsic motivation, and so in certain cases one can only infer from observed behavior that intrinsic motivation was crowded out by extrinsic incentives. Our results suggest a similar phenomenon can manifest as a product of specific incentivization schemes that encourage under-exploration and suboptimal choice. Once incentives are removed, learners may favor disengaging from the no longer profitable suboptimal options, leading to results that mimic a crowding out effect. Importantly, our alternative explanation does not need to rely on motivational and intrinsic factors but is driven solely by learning of the incentive structure. Future research can disentangle motivational crowding out effects and specific learning traps produced by incentivization programs that lead to under-exploration of beneficial but challenging outcomes (see also Eisenberger & Cameron, 1996).

Note that our results are particularly relevant to Try environments in which finding extremely good options rarely happens. If great options are relatively easy to find, previous studies suggest people would be more risk seeking and try much harder to find such options (even assuming larger opportunity costs; see Ludvig et al., 2014a, b, 2018; Madan, et al., 2019). This also suggests that to tackle behavioral problems, interventions can focus on increasing the probability of finding great options (e.g., improving the level of education), increasing in turn people’s effort to try and find such options.

The current research contributes to our understanding of incentivization strategies and their impact on behavior. Our results highlight the potential of financial incentives in promoting exploration of beneficial but challenging alternatives, while underscoring the need to address options that are suboptimal but easier to attain. Future studies can clarify how interventions interact with contextual factors to develop strategies that avoid suboptimal behavior and learning traps, ensuring sustained engagement with rewarding challenges. As the current study suggests, the most promising programs are those focusing on encouraging exploration of challenging but ultimately beneficial options, and/or reducing opportunity costs implied by avoiding these options.

## Supplementary information

Below is the link to the electronic supplementary material.

Supplementary file1 (DOCX 1688 KB)

## Data Availability

All data, simulation code and power analyses are available online (https://osf.io/w3vea). See preregistration (https://aspredicted.org/H5V_77Q).
