# Supplementary material for: Don’t Give-Up: Why some intervention schemes encourage suboptimal behavior
Source: Psychon Bull Rev. 2024 Jul 23;32(1):363–72. doi: 10.3758/s13423-024-02537-w (PMC11836215; doi:10.3758/s13423-024-02537-w)
Supplement: Supplementary file 1 — Supplementary file1 (DOCX 1688 KB) [file 13423_2024_2537_MOESM1_ESM.docx]

**Appendix A**

Table A1 presents the simulation results of different solution strategies for the “Try or Give up” dilemma employed in our study.

**Table A1.** *Simulated solutions for Baseline game payoff matrix and Optimal benchmark.*

| Strategies | Simulation results:  Expected mean outcome per trial (in points) |
| --- | --- |
| *Search for +14 outcomes in Try matrix until trial t. If found by trial t, exploit outcome to the end of round. If not found, switch at trial t+1 to Give-Up matrix, then find and exploit +2 outcome.* | |
| *t* = 1 | 3.70 |
| *t* = 2 | 4.32 |
| *t* = 3 | 4.82 |
| *t* = 4 | 5.19 |
| *t* = 5 | 5.46 |
| *t* = 6 | 5.66 |
| *t* = 7 | 5.79 |
| *t* = 8 | 5.86 |
| ***t* = 9 (Optimal strategy)** | **5.89** |
| *t* = 10 | 5.88 |
| *t* = 11 | 5.85 |
| *Other strategies* |  |
| Search for, and exploit once found, the best outcome (+2) in the Give-Up matrix (never switch) | 1.62 |
| Search for, and exploit once found, the best outcome (+14) in the Try matrix (never switch) | 5.81 |
| Randomly choose one matrix, and then randomly choose keys in that matrix only | 0.6 |
| Randomly choose keys from both matrices | 0.3 |
|  |  |

*Note.* Each simulation was run one million times and used the task parameters explained in the Methods section above. Simulated results denote the mean point amount expected per trial in the baseline rounds of the study, conditional on the strategy used. Simulation code can be found at: https://osf.io/w3vea.

**Appendix B**

**
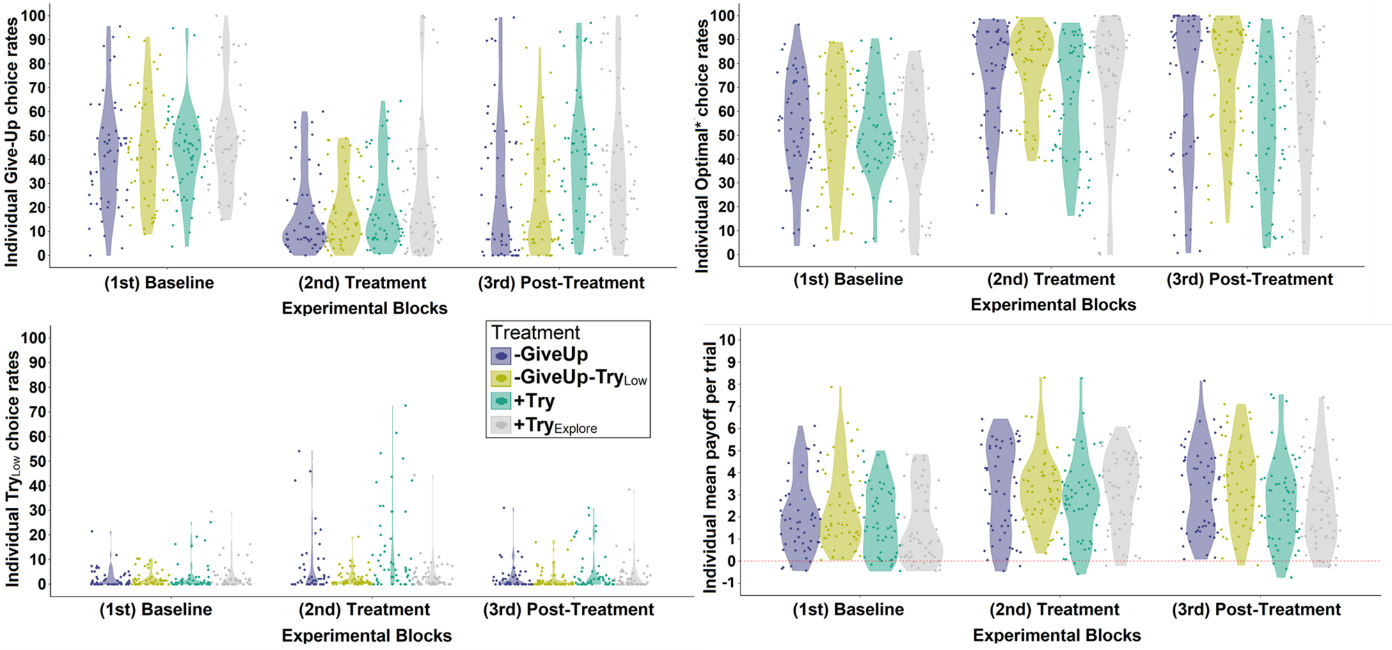
Figure B1.** *Individual differences* *summarized over the main dependent variables.*

**(D)**

**(C)**

**(B)**

**(A)**

*Note.* Each figure presents individual-level average results for each main dependent variable. Each dot represents the result of one participant in either the 1^st^, 2^nd,^ or 3^rd^ experimental block. Panel A shows individual-level (%) Give-Up rates across the three experimental blocks, Panel B shows Optimal choice rates (i.e., searching for, and then exploiting, +14 outcomes), Panel C shows ${Try}_{Low}$ and Panel D shows average per trial payoffs. Error bars show 95% Confidence Intervals.

**Figure B2.** *Main dependent variables* *as a function of Trial (across rounds).*


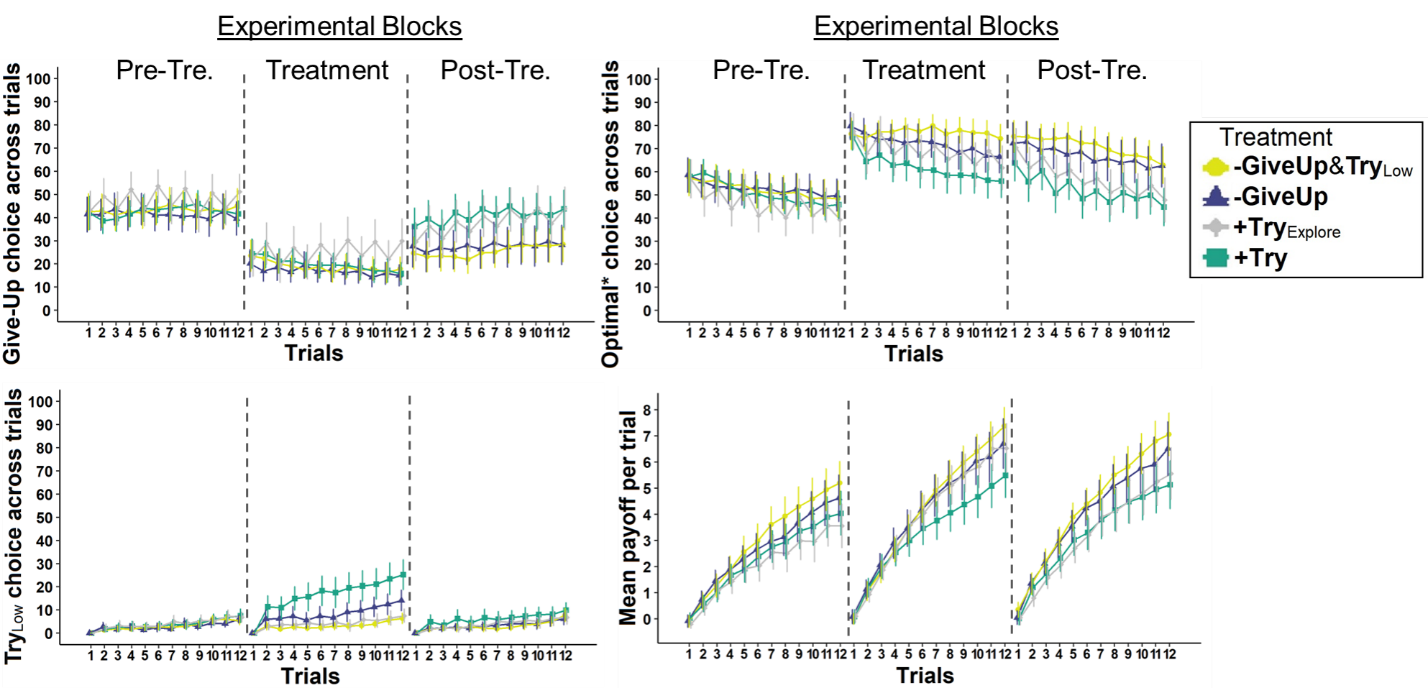


**(A)**

**(B)**

**(C)**

**(D)**

*Note.* Each figure presents average results for each main dependent variable summarized as a function of trial number (across rounds). Pre-Tre. And Post-Tre. titles denote the Pre-Treatment (Baseline) and Post-Treatment experimental Blocks, respectively. Panel A shows mean (%) Give-Up rates across the 12 trials in each of the three experimental blocks. Panel B shows Optimal choice rates (i.e., searching for, and then exploiting, +14 outcomes), Panel C shows ${Try}_{Low}$ rates (i.e., exploiting the +1 outcome in the Try matrix) and Panel D shows average payoffs per trial. Error bars show 95% Confidence Intervals.
